# Triglyceride to high-density lipoprotein cholesterol ratio and total cholesterol to high-density lipoprotein cholesterol ratio and risk of benign prostatic hyperplasia in Chinese male subjects

**DOI:** 10.3389/fnut.2022.999995

**Published:** 2022-10-03

**Authors:** Chen Zhu, Juan Wu, Yixian Wu, Wen Guo, Jing Lu, Wenfang Zhu, Xiaona Li, Nianzhen Xu, Qun Zhang

**Affiliations:** Department of Health Promotion Center, The First Affiliated Hospital with Nanjing Medical University, Nanjing, China

**Keywords:** benign prostatic hyperplasia, dyslipidemia, lipid ratios, triglycerides, high-density lipoprotein cholesterol

## Abstract

**Background:**

Lipid metabolism disorders contribute to the risk factor of prostatic hyperplasia. Lipid ratios have also attracted a lot of attention. Yet, research about the correlation of lipid ratios with prostatic hyperplasia is limited. Hence, the aim of this study was to investigate the association of lipid ratios with the risk of benign prostatic hyperplasia (BPH) in Chinese male subjects.

**Methods:**

Healthy men who underwent routine health check-ups from January 2017 to December 2019 were recruited. Twenty-four thousand nine hundred sixty-two individuals were finally enrolled in this research. Binary logistic regression analysis was performed to investigate the relationship between lipid ratios and BPH in Chinese adults.

**Results:**

After health examinations for more than 2 years, 18.46% of subjects were ascertained as incident BPH cases. Higher age, body mass index (BMI), prostate-specific antigen (PSA), triglycerides (TGs), low-density lipoprotein cholesterol (LDL-C), triglyceride to high-density lipoprotein cholesterol (TG/HDL-C) ratio, total cholesterol to high-density lipoprotein cholesterol (TC/HDL-C) ratio, and lower high-density lipoprotein cholesterol (HDL-C) were significantly associated with BPH risk, while total cholesterol (TC) was not significant. When quartiles of TG/HDL-C and TC/HDL-C were analyzed in multivariable model, higher TG/HDL-C and TC/HDL-C were associated with a risk of BPH (odds ratio [OR] = 2.11; 95% confidence interval [CI]: 1.89, 2.36; *P*-trend < 0.001; and OR = 1.67; 95% CI: 1.50, 1.85; *P*-trend < 0.001, respectively). In addition, stratified analyses based on the general population exhibited that with increasing age (≥35 years) the relationship of TG/HDL-C ratio with BPH risk was dominantly positive (all *P*-trend < 0.001, *P*-interaction = 0.001), and significant associations were also found in blood pressure strata and FBG strata (all *P*-trend < 0.001), except men with BMI ≥ 28 kg/m^2^ were slightly weakened (OR = 2.01, 95% CI: 1.41, 2.85; *P*-trend = 0.04). Moreover, there were significant associations between quartiles of TC/HDL-C and the risk of BPH was observed mainly in age 55–64 years, BMI 18.5–23.9 Kg/m^2^, blood pressure strata, and FBG strata. However, the *P*-value for a linear trend among those with BMI ≥ 28 Kg/m^2^ in which participants at the highest quartile of TC/HDL-C had an OR of 1.45 (95% CI: 1.09, 1.93) was 0.594. Additionally, higher TG/HDL-C ratio (≥0.65) may be a risk factor for BPH in China adults of different age decades (≥35 years) with normal TG and HDL-C.

**Conclusions:**

TG/HDL-C and TC/HDL-C were associated with BPH risk, TG/HDL-C was a powerful independent risk factor for BPH in Chinese adults, and higher TG/HDL-C ratio should be valued in male subjects with normal TG and HDL-C levels.

## Introduction

With the development of society and economy, the rapidly aging problem is prominent, especially in China. BPH is a common disease in elderly men and has become an important public health challenge (1). BPH, also known as benign prostatic obstruction, is a histologic diagnosis that refers to non-malignant proliferation and unregulated growth of the epithelial cells and stromal cells (2). Lower urinary tract symptoms (LUTSs) secondary to BPH (BPH/LUTS) include urinary retention, bifurcation of urination, and pause in urine stream (3, 4), among others. Thus, if the control of BPH has been ineffective, the probability is that LUTS will rise accordingly. Furthermore, BPH increased from 2.17 to 31.11% within the age ranging from 40 to 80 or older (5), and kidneys may become damaged over time as the symptoms of BPH/LUTS intensify. China’s seventh national census shows that the proportion of the population aged over 60 years is 18.70% and increased by 5.44% than 10 years ago (6). This is undoubtedly a powerful reserve for the prostatic hyperplasia population. In addition to that, the epidemiological status of BPH, high prevalence of LUTS/BPH, the severity of the disease, treatment methods, etc. (7–9), all of which may lead to the cost of illness (COI) becoming a major challenge in future. Hence, strengthening the health management and early intervention of BPH will significantly reduce the burden on the elderly population and society.

Currently, the pathogenesis of BPH still remains unclear, except that age is the strongest factor. Various studies (2, 3, 10) have been devoted to revealing the underlying molecular etiology of this common disease, such as observational study, genetic analysis, and biochemical and histochemical analyses. Fujita et al. pointed out that the white blood cell (WBC) count and the neutrophil (NEUT) count were correlated with prostate volume (11), and De Nunzio et al. reported that the mediators of inflammation played a role in the course of the development and progression of BPH (12). Insufficient physical activity, poor eating habits, and obesity are the most common causes of metabolic syndrome (MetS). This phenomenon has triggered focus attention to the role of the Mets in the life cycle. Data have shown a possible relationship between MetS and BPH, such as hyperlipemia, adiposity, hyperglycemia, hypertension (13–16). Especially low HDL-C and hypertriglyceridemia levels were proved to be closely correlated with BPH (17–19). Inflammatory response may be the pathogenesis of BPH caused by dyslipidemia (20). Although, the association of MetS with prostatic hyperplasia has been researched for many years. An in-depth description of the role of lipid components in BPH remains promising to open new perspectives into the occurrence and progression of disease.

BPH risk is associated with an aberrant lipid profile in many races. Previous research in Turkish male subjects showed that TG/HDL-C was significantly related to prostate volume (21). However, the TC/HDL-C ratio was not assessed, and the correlation between TG/HDL-C and prostatic hyperplasia was also not further analyzed in different age decades and in Turkish adults with normal TG and HDL-C levels. To deeply elucidate the relationships of lipid ratios with BPH in the general population and in participants only with normal TG and HDL-C, and to investigate the association between BPH and lipid ratios in different age decades, we conducted a survey using a population-based dataset in China.

## Materials and methods

### Study population

**FIGURE 1:**
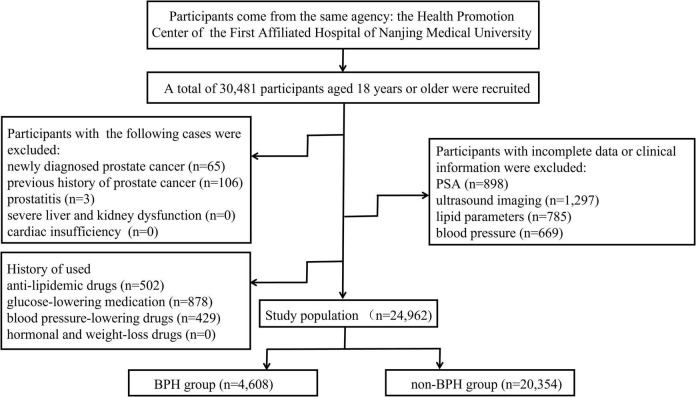
Schematic diagram of the selection of study objects.

A total of 30,481 health screening population who visited the Health Promotion Center of the First Affiliated Hospital of Nanjing Medical University were recruited for this study between January 2017 and December 2019. The participants were predominantly from China, and all of the subjects were classified into BPH group and non-BPH group. The gold standard for prostate examination is prostate pathological cytology; however, it is not recommended for large-scale population studies due to its invasiveness, the risk of complications, and high cost. Expensive MRI examination has also not found an increasingly wide utilization in detecting prostate except for patients with suspected prostate cancer. Herein, the criteria for inclusion in this trial are abdominal ultrasound imaging with no suspicion of prostate cancer, and the volume of the prostate is determined by height (cm) × width (cm) × length (cm) × π/6 based on the abdominal ultrasound measurements, the normal volume is 20–30 mL, the secretion of PSA is influenced by prostate volume size, so there is no special requirement for PSA levels (22). It is noteworthy that the individuals enrolled in the group had undergone health examinations for more than 2 years, which was helpful to exclude inappropriate candidates. The participants with prostatitis, severe liver and kidney dysfunction, cardiac insufficiency, and taking anti-lipidemic drugs or glucose-lowering medication, hormonal and weight-loss drugs, blood pressure-lowering drugs were excluded, as were those subjects with incomplete information. Finally, 24,962 participants were enrolled in this study (Figure 1).

All participants gave written consent to participate. The protocol was approved by the Ethics Committee of the First Affiliated Hospital of Nanjing Medical University (ethics committee approval number: 2019-SR-478), also based on the Declaration of Helsinki (as revised in 2013).

### Anthropometric and laboratory measurements

Data collection was performed by well-trained staff from the above hospital. BMI was calculated by dividing the weight (kg) by the square of height (m), according to the statement of Working Group on Obesity in China, BMI greater than or equal to 28 kg/m^2^ is defined as obesity (23). The electronic sphygmomanometer was used to measure blood pressure for three times. Blood samples were collected from subjects after being fasted overnight for more than 8 h. Biochemical parameters like PSA, fasting blood glucose (FBG), TG, TC, LDL-C, and HDL-C were assessed with the Chemistry Analyzer AU5800 (Olympus Corporation, Tokyo, Japan).

### Statistical analysis

P–P plots were used to determine the normal distribution of the variables. Continuous variables were presented as mean ± SD (standard deviations) and compared using an independent *t*-test. Mann–Whitney *U* test was used to analyze the skewed variables represented as the median and interquartile range (IQR).

The associations of lipid ratios with BPH were further determined by binary logistic regression analysis. In model 1, age and PSA were adjusted. Model 2 was based on Model 1 by adding BMI and FBG. WBC, NEUT, and hypertension were additionally calibrated in Model 3. These gradual multivariable models were carried out to determine whether the relationships of lipid ratios with BPH were robust. Linear trends were performed with the use of quartiles of the lipid ratios as a continuous variable by calculating the corresponding median TG/HDL-C and TC/HDL-C for each quartile, respectively.

Additionally, stratified analysis was further conducted on all participants. To decrease the impact of outliers, the subjects were stratified into five age groups (<35, 35–44, 45–54, 55–64, ≥65 years), three BMI groups (18.5–23.9, 24–27.9, ≥28 Kg/m^2^), two blood pressure groups (normal, hypertension), and two FBG groups (<6.1, ≥6.1 mmol/L), and each subgroup was classified into quartiles according to TG/HDL-C and TC/HDL-C levels, with the lowest quartile being the reference group. The stratified analysis of TG/HDL-C on the risk of BPH was also performed among male subjects with normal TG and HDL-C levels by age (<35, 35–44, 45–54, 55–64, ≥65 years), BMI (<18.5, 18.5–23.9, 24–27.9, ≥28 Kg/m^2^), blood pressure levels (normal and hypertension), and FBG (<6.1 and ≥6.1 mmol/L). Model a: WBC, NEUT, PSA, hypertension, FBG, and BMI were adjusted. Model b: age, WBC, NEUT, PSA, hypertension, and FBG were adjusted. Model c: age, WBC, NEUT, PSA, FBG, and BMI were adjusted. Model d: age, WBC, NEUT, PSA, hypertension, and BMI were adjusted. The likelihood ratio test (LRT) was used to investigate the interactions between lipid ratios and other variables.

All of the data were carried out using SPSS version 23.0 (IBM, Chicago, IL, USA). A *P-*value < 0.05 (two-sided) was considered statistically significant. The significance of multicollinearity phenomena in multivariate regression analyses was assessed by calculating the tolerance and variance inflation factor (VIF).

## Results

Baseline characteristics of 24,962 participants included in this study were displayed in Table 1. The mean age, TC, HDL-C, BMI, and the median TG, PSA of the study population were 46.15 ± 13.24 years, 5.06 ± 0.8 mmol/L, 1.22 ± 0.24 mmol/L, 25.04 ± 2.96 kg/m^2^, 1.45 mmol/L, 0.88 ng/ml, respectively.

**TABLE 1:** Clinical and biochemical parameters of the study population.

Characteristics	Overall (*n* = 24,962)	Non-BPH (*n* = 20,354)	BPH (*n* = 4,608)	*P*-value
Age (years)	46.15 ± 13.24	44.14 ± 12.69	55.03 ± 11.87	<0.001
WBC (10^9^/L)	6.09 ± 1.23	6.08 ± 1.2	6.15 ± 1.36	0.001
NEUT (10^9^/L)	3.49 ± 0.94	3.47 ± 0.92	3.57 ± 1.02	<0.001
FBG (mmol/L)	5.32 (5–5.76)	5.31 (4.99–5.71)	5.41 (5.03–5.98)	<0.001
TG (mmol/L)	1.45 (1.04–2.07)	1.42 (1.02–2.03)	1.59 (1.14–2.24)	<0.001
TC (mmol/L)	5.06 ± 0.89	5.05 ± 0.87	5.07 ± 0.97	0.318
HDL-C (mmol/L)	1.22 ± 0.24	1.23 ± 0.25	1.17 ± 0.19	<0.001
LDL-C (mmol/L)	3.2 ± 0.64	3.2 ± 0.62	3.17 ± 0.72	0.001
TG/HDL-C	1.22 (0.8–1.88)	1.18 (0.77–1.83)	1.36 (0.94–2.07)	<0.001
TC/HDL-C	4.25 ± 0.94	4.22 ± 0.93	4.41 ± 0.97	<0.001
PSA (ng/mL)	0.88 (0.61–1.33)	0.84 (0.59–1.24)	1.13 (0.73–1.83)	<0.001
SBP (mmHg)	128 (118–139)	127 (118–138)	132 (121–144)	<0.001
DBP (mmHg)	80.64 ± 10.8	80.22 ± 10.78	82.51 ± 10.7	<0.001
BMI (kg/m^2^)	25.04 ± 2.96	24.95 ± 2.98	25.43 ± 2.86	<0.001

BPH, benign prostatic hyperplasia; WBC, white blood cell; NEUT, neutrophil; FBG, fasting blood glucose; TG, triglyceride; TC, total cholesterol; HDL-C, high-density lipoprotein cholesterol; LDL-C, low-density lipoprotein cholesterol; TG/HDL-C, triglyceride/high-density lipoprotein cholesterol; TC/HDL-C, cholesterol/high-density lipoprotein cholesterol; PSA, prostate-specific antigen; SBP, systolic blood pressure; DBP, diastolic blood pressure; BMI, body mass index.

There were 18.46% participants with BPH and had higher levels of age, NEUT, FBG, TG, LDL-C, PSA, SBP, DBP, BMI, but lower HDL-C levels. In addition, the values of TG/HDL-C and TC/HDL-C were also notably different between the BPH group and non-BPH group. However, no significant difference was found in TC between the two groups.

The associations of TG/HDL-C and TC/HDL-C ratios with BPH were evaluated in models 1–3 as shown in Table 2. According to TG/HDL-C and TC/HDL-C levels, 24,962 participants were classified into quartiles, respectively. In the unadjusted model, the ORs (95% CI) of BPH across increasing quartiles of TG/HDL-C and TC/HDL-C were 1.00, 1.59 (1.44–1.76), 1.82 (1.65–2.00), 1.98 (1.80–2.18), and 1.00, 1.21 (1.10–1.33), 1.47 (1.34–1.61), and 1.58 (1.44–1.73) (all *P*-trend < 0.001), respectively. Higher TG/HDL-C and TC/HDL-C were associated with a significantly high risk of BPH when adjusting for age and PSA; the ORs (95% CI) were 2.25 (2.03–2.50) and 1.83 (1.65–2.02) (all *P*-trend < 0.001), respectively. The associations were still significant after further adjustment for potential confounders (WBC, NEUT, FBG, BMI, and hypertension); the ORs (95% CI) were 2.11 (1.89–2.36), and 1.67 (1.50–1.85) (all *P*-trend < 0.001), respectively.

**TABLE 2:** Logistic regression models assessing the associations of lipid ratios with BPH.

	Q1	Q2	Q3	Q4	*P*-trend^1^
**TG/HDL-C**					
Median	0.61	1	1.49	2.59	
Unadjusted	1.00 (Ref)	1.59 (1.44–1.76)***	1.82 (1.65–2.00)***	1.98 (1.80–2.18)***	
Model 1	1.00 (Ref)	1.65 (1.49–1.84)***	1.94 (1.75–2.16)***	2.25 (2.03–2.50)***	<0.001
Model 2	1.00 (Ref)	1.60 (1.44–1.79)***	1.85 (1.66–2.06)***	2.12 (1.90–2.36)***	<0.001
Model 3	1.00 (Ref)	1.60 (1.44–1.78)***	1.85 (1.66–2.06)***	2.11 (1.89–2.36)***	<0.001
**TC/HDL–C**					
Median	3.18	3.92	4.53	5.36	
Unadjusted	1.00 (Ref)	1.21 (1.10–1.33)***	1.47 (1.34–1.61)***	1.58 (1.44–1.73)***	
Model 1	1.00 (Ref)	1.35 (1.22–1.50)***	1.68 (1.52–1.86)***	1.83 (1.65–2.02)***	<0.001
Model 2	1.00 (Ref)	1.3 (1.17–1.44)***	1.58 (1.42–1.75)***	1.68 (1.52–1.87)***	<0.001
Model 3	1.00 (Ref)	1.30 (1.17–1.44)***	1.57 (1.42–1.74)***	1.67 (1.50–1.85)***	<0.001

Model 1: adjusted for age, PSA.

Model 2: adjusted for Model 1 variables in addition to BMI, FBG.

Model 3: adjusted for Model 2 variables in addition to WBC, NEUT, hypertension. Ref, reference; OR, odds ratio; CI, confidence interval; TG/HDL-C, triglyceride/high-density lipoprotein cholesterol; TC/HDL-C, cholesterol/high-density lipoprotein cholesterol; WBC, white blood cell; NEUT, neutrophil; FBG, fasting blood glucose; HDL-C, high-density lipoprotein cholesterol; LDL-C, low-density lipoprotein cholesterol; BMI, body mass index.

***P-value < 0.001.

^1^Test for trend based on the variable containing a median value for each quartile.

To further investigate the associations of TG/HDL-C and TC/HDL-C with BPH risk, stratified multivariable logistic regression analyses were conducted to calculate the adjusted ORs (95% CI). Taking into account the study power of the stratified analyses, each subgroup is categorized by age strata, BMI strata, blood pressure strata, and FBG strata, respectively. A significant positive correlation between TG/HDL-C and risk of BPH was found in 11 different subgroups except for men aged below 35 years (Table 3), and a linear trend among quartiles of TG/HDL-C and BPH risk in these 11 subgroups was also significant. Moreover, only six different subgroups (age 55–64 years, BMI 18.5–23.9 Kg/m^2^, normal blood pressure or hypertension, FBG < 6.1 or ≥ 6.1 mmol/L) showed that the relationship of TC/HDL-C with BPH risk was significant. The multivariate-adjusted ORs (95% CI) of BPH in men aged 65 years and above or BMI 18.5–23.9 Kg/m^2^ or normal blood pressure or FBG < 6.1 mmol/L for the highest quartile of TG/HDL-C compared with the lowest were 2.04 (1.55–2.68), 2.61 (2.15–3.16), 2.21 (1.92–2.55), and 2.09 (1.85–2.37), while the ORs (95% CI) of BPH were 1.52 (1.19–1.95), 2.24 (1.86–2.69), 1.56 (1.36–1.78), and 1.55 (1.38–1.75) for TC/HDL-C. Additionally, the interactions of TG/HDL-C with age strata, BMI strata, and FBG strata were all significant, while a test of interaction between TG/HDL-C with blood pressure strata on BPH was not (*P*-interaction = 0.379). And the siginificant interaction effects were also detected between TC/HDL-C and age strata, BMI strata, FBG strata, and blood pressure strata.

**TABLE 3:** ORs of BPH by quartiles of TG/HDL-C and TG/HDL-C stratified by age, BMI, blood pressure, and FBG.

	Q1	Q2	Q3	Q4	*P*-trend^1^	*P*-interaction
		OR (95% CI)	OR (95% CI)	OR (95% CI)		
**TG/HDL-C**						
Age^a^						0.001
<35	1.00 (Ref)	1.31 (0.81–2.12)	1.09 (0.63–1.87)	1.40 (0.81–2.45)	0.126	
35–44	1.00 (Ref)	1.36 (1.04–1.79)*	1.64 (1.26–2.14)***	1.69 (1.28–2.22)***	<0.001	
45–54	1.00 (Ref)	1.53 (1.27–1.83)***	1.71 (1.42–2.05)***	1.88 (1.56–2.25)***	<0.001	
55–64	1.00 (Ref)	1.56 (1.26–1.93)***	1.71 (1.38–2.11)***	2.02 (1.63–2.51)***	<0.001	
≥65	1.00 (Ref)	1.64 (1.30–2.08)***	1.94 (1.51–2.48)***	2.04 (1.55–2.68)***	<0.001	
BMI^b^						<0.001
18.5–23.9	1.00 (Ref)	1.74 (1.48–2.05)***	2.14 (1.80–2.55)***	2.61 (2.15–3.16)***	<0.001	
24–27.9	1.00 (Ref)	1.47 (1.25–1.72)***	1.67 (1.43–1.95)***	1.90 (1.63–2.22)***	<0.001	
≥28	1.00 (Ref)	1.54 (1.06–2.23)*	1.75 (1.22–2.50)**	2.01 (1.41–2.85)***	0.04	
Blood pressure^c^						0.379
Normal	1.00 (Ref)	1.7 (1.49–1.94)***	1.88 (1.64–2.15)***	2.21 (1.92–2.55)***	<0.001	
Hypertension	1.00 (Ref)	1.44 (1.20–1.73)***	1.77 (1.48–2.11)***	1.90 (1.58–2.28)***	<0.001	
FBG^d^						0.013
<6.1	1.00 (Ref)	1.65 (1.47–1.86)*	1.89 (1.67–2.12)***	2.09 (1.85–2.37)***	<0.001	
≥6.1	1.00 (Ref)	1.42 (1.09–1.85)**	1.65 (1.28–2.14)***	1.97 (1.53–2.54)***	<0.001	
**TC/HDL-C**						
Age^a^						<0.001
<35	1.00 (Ref)	0.54 (0.33–0.9)*	0.73 (0.45–1.2)	0.7 (0.41–1.2)	0.755	
35–44	1.00 (Ref)	1.13 (0.87–1.48)	1.41 (1.09–1.82)**	1.3 (1–1.7)	0.023	
45–54	1.00 (Ref)	1.19 (1–1.41)	1.36 (1.15–1.62)***	1.54 (1.3–1.83)***	<0.001	
55–64	1.00 (Ref)	1.38 (1.12–1.71)**	1.7 (1.38–2.09)***	1.65 (1.34–2.03)***	<0.001	
≥65	1.00 (Ref)	1.23 (0.98–1.55)	1.45 (1.15–1.84)**	1.52 (1.19–1.95)***	<0.001	
BMI^b^						<0.001
18.5–23.9	1.00 (Ref)	1.48 (1.26–1.75)***	1.69 (1.42–2.02)***	2.24 (1.86–2.69)***	<0.001	
24–27.9	1.00 (Ref)	1.12 (0.96–1.3)	1.46 (1.26–1.69)***	1.45 (1.26–1.68)***	<0.001	
≥28	1.00 (Ref)	1.17 (0.85–1.6)	1.39 (1.03–1.87)**	1.45 (1.09–1.93)**	0.594	
Blood pressure^c^						0.002
Normal	1.00 (Ref)	1.26 (1.11–1.44)***	1.48 (1.3–1.68)***	1.56 (1.36–1.78)***	<0.001	
Hypertension	1.00 (Ref)	1.29 (1.08–1.54)**	1.69 (1.42–2)***	1.78 (1.5–2.12)***	<0.001	
FBG^d^						0.002
<6.1	1.00 (Ref)	1.25 (1.11–1.4)***	1.6 (1.43–1.8)***	1.55 (1.38–1.75)***	<0.001	
≥6.1	1.00 (Ref)	1.37 (1.08–1.74)**	1.37 (1.09–1.73)**	1.86 (1.49–2.33)***	0.005	

Model a: WBC, NEUT, FBG, PSA, hypertension, BMI.

Model b: age, WBC, NEUT, FBG, PSA, hypertension.

Model c: age, WBC, NEUT, FBG, PSA, BMI.

Model d: age, WBC, NEUT, PSA, hypertension, BMI.

Ref, reference; OR, odds ratios; CI, confidence interval; TG/HDL-C, triglyceride/high-density lipoprotein cholesterol; TC/HDL-C, cholesterol/high-density lipoprotein cholesterol; WBC, white blood cell; NEUT, neutrophil; FBG fasting blood glucose; HDL-C, high-density lipoprotein cholesterol; LDL-C, low-density lipoprotein cholesterol; BMI, body mass index.

*P-value < 0.05.

**P-value < 0.01.

***P-value < 0.001.

^1^Test for trend based on the variable containing the median value for each quartile.

**FIGURE 2:**
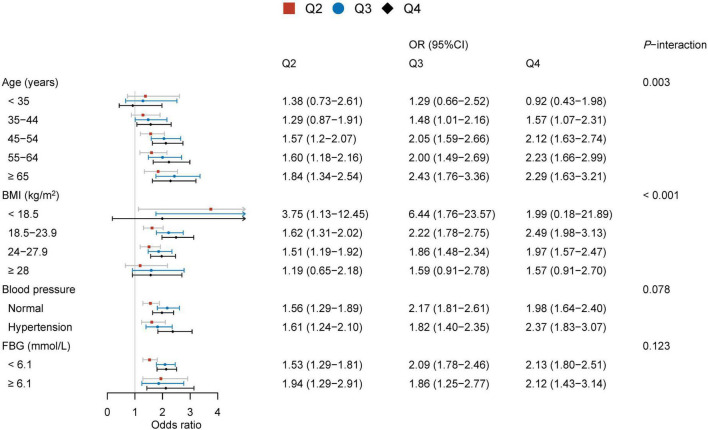
Associations between TG/HDL-C and BPH in subjects with normal TG and HDL-C levels.

Additionally, 13,983 subjects with normal TG (<1.7 mmol/L) and HDL-C (≥1.0 mmol/L) levels were also divided into strata based on defining features (age, BMI, blood pressure, FBG). The quartiles of TG/HDL-C were calculated [Q1 (<0.65), Q2 (0.65–0.84), Q3 (0.85–1.09), and Q4 (≥ 1.1)] in these participants. Based on the estimated effects of other determinants, binary logistic regression models were used to determine the association between TG/HDL-C and BPH. The logistic analyses showed that TG/HDL-C and BPH were statistically significant except in men aged ≤ 44 years and obese subjects (Figure 2). Again, significant interaction effects were observed between TG/HDL-C and age strata and TG/HDL-C and BMI strata on BPH (*P*-interaction = 0.003, *P*-interaction < 0.001, respectively), while no interactions were observed between TG/HDL-C and blood pressure strata and TG/HDL-C and FBG strata in participants.

In all logistic regression models, the test of multicollinearity suggested no collinearity with a range of the variance inflation factor (VIF) between 1.08 and 4.56 and the tolerance between 0.22 and 0.93.

## Discussion

Benign prostatic hyperplasia is one of the most common chronic non-communicable diseases in aging male subjects, and the burden of BPH is now increasing. In this research, the data showed that there were significant associations between TG/HDL-C and TC/HDL-C ratios and BPH risk in a large Chinese population. TG/HDL-C was a powerful independent risk factor for BPH, even though individuals did not have abnormal TG and HDL-C. These findings indicate that the risk of BPH is affected by the value of TG/HDL-C, regardless of the levels of TG and HDL-C. Thus, male subjects with higher TG/HDL-C should be given health guidance or lipid-lowering therapy to reduce the BPH risk.

Clinical and epidemiology studies have already documented that lipid ratio is a useful lipid parameter associated with diseases. For instance, Sánchez-Íñigo et al. (24) evidenced that triglycerides-related variables had the advantage to evaluate the risk of developing hypertension in the Spanish population. Wei et al. (25) found that a high concentration of lipid ratios worked synergistically with hypertension to affect the incidence of ischemic stroke. A lot of research pointed out that TG/HDL-C was favorable to identify increased urinary albumin-to-creatinine ratio, insulin resistance, diabetes risk, incident hypertension, and cardiovascular events (26–31). By far, there has been little study on the relationship between lipid ratios and BPH, except for the Turkish research in which 400 patients were enrolled (21). Yet, only TG/HDL-C was included in the Turkish analysis, and the sample size was of course very small. Our study suggested that TG/HDL-C and TC/HDL-C were related to BPH risk. Notably, the span of TG and HDL-C levels in the Chinese subjects exceeded that of the Turkish sample, and the age groups covered were also relatively broad. These guarantee sufficient power in the investigation of complicated interactions between TG/HDL-C and TC/HDL-C and other confounding factors.

Age is an important risk factor for the occurrence of BPH. As expected, significant positive relationships were observed in TG/HDL-C and BPH in different age decades except for men aged below 35 years, in which the power of the correlation between TG/HDL-C ratio and BPH increased with increasing subjects’ age. Men aged 35 years or over with a normal range of TG and HDL-C also need to take into account the risk of BPH from the higher TG/HDL-C ratio (≥0.65). However, a robust association between TC/HDL-C and the risk of BPH was not found in the stratified analysis by age.

In the stratified multivariable logistic regression analysis by blood pressure and FBG, the relationships between the ratios of TG or TC to HDL-C and BPH were all significant. However, an interesting phenomenon was observed between lipid ratios and BPH across BMI subgroups, the effect of TG/HDL-C on BPH risk was slightly attenuated with increasing BMI values, and the linear trend was not significant between the highest quartile of TC/HDL-C and the risk of BPH. Moreover, the correlation between TG/HDL-C and BPH was not significant in obese individuals whose TG and HDL-C were both within the normal range. This may be related to the increased autonomic hyperactivity caused by increased BMI, which affects BPH growth (32). So the effect of lipid ratios on BPH risk in men with increasing BMI may be blunted by this mechanism.

At present, the growing demand for health services lies in the fact that the incidence of non-communicable diseases is increasing and showing a trend in youth. It is necessary to meet the growing health needs of the people. The purpose of this article was to identify the relationships between lipid ratios and BPH that should be considered in practical work to improve health management services. And it should be used more widely to recognize high-risk populations. This is also clinically important because dyslipidemia is treatable.

### Study strengths and limitations

In this research, the major strengths are (1) the cohort is based on a large population sample size of 24,962 participants, (2) the effects of TG/HDL-C and TC/HDL-C on BPH risk were both analyzed, (3) further analyze the association between TG/HDL-C and BPH in adults with normal TG and HDL-C levels, and (4) this is the first study to show these findings in China. This study offers reliable and powerful information for further studies about the relationships of disease with blood lipids. Nevertheless, there are several limitations to the study. First, the diagnosis of BPH is mainly based on transabdominal sonography, not prostate biopsy, and might have introduced inaccuracies into this study. Second, the current study was mainly conducted on the Chinese male population; similar results may not be available for other races. Third, this single-institution study may lead to selection bias and limit its generalizability, unadjusted variables including education, diet, regional environment, work situation, marital status, etc. also may result in potential bias and limit the validity of the results. Future research should consider the potential effects of these confounding factors more carefully. In addition, due to the inherent character of the cross-sectional study, this study can only assess the relationship rather than causality.

## Conclusion

In this study, the TG/HDL-C and TC/HDL-C ratios are correlated with the risk of BPH in the Chinese population. TG/HDL-C was a powerful independent risk factor for BPH. Furthermore, a higher TG/HDL-C value is useful to assess BPH risk in adults with normal TG and HDL-C levels. Hence, more attention should be paid to those with a higher TG/HDL-C in the health management process, and lifestyle modifications or lipid-lowering agents to help prevent BPH risk in middle-aged and older adults.

## Data availability statement

The original contributions presented in this study are included in the article/supplementary material, further inquiries can be directed to the corresponding author.

## Ethics statement

The studies involving human participants were reviewed and approved by Ethics Committee of the First Affiliated Hospital of Nanjing Medical University (Ethics Committee approval number: 2019-SR-478). The patients/participants provided their written informed consent to participate in this study.

## Author contributions

CZ and QZ contributed to the conception, design, data analysis, and drafting of the manuscript. YW, JW, and WG participated in the study design. JL, WZ, XL, and NX contributed to the conduct of the study and data collection. All authors read and approved the final manuscript.
